# Spontaneous head-movements improve sound localization in aging adults with hearing loss

**DOI:** 10.3389/fnhum.2022.1026056

**Published:** 2022-10-13

**Authors:** Elena Gessa, Elena Giovanelli, Domenico Spinella, Grégoire Verdelet, Alessandro Farnè, Giuseppe Nicolò Frau, Francesco Pavani, Chiara Valzolgher

**Affiliations:** ^1^Center for Mind/Brian Sciences - CIMeC, University of Trento, Rovereto, Italy; ^2^“S. Maria del Carmine” Hospital, Rovereto, Italy; ^3^Integrative, Multisensory, Perception, Action and Cognition Team-IMPACT, Centre de Recherche en Neuroscience de Lyon, University Lyon 1, Lyon, France; ^4^Neuro-immersion, Centre de Recherche en Neuroscience de Lyon, Lyon, France

**Keywords:** aging, sound localization, head movements, hearing loss, virtual reality

## Abstract

Moving the head while a sound is playing improves its localization in human listeners, in children and adults, with or without hearing problems. It remains to be ascertained if this benefit can also extend to aging adults with hearing-loss, a population in which spatial hearing difficulties are often documented and intervention solutions are scant. Here we examined performance of elderly adults (61–82 years old) with symmetrical or asymmetrical age-related hearing-loss, while they localized sounds with their head fixed or free to move. Using motion-tracking in combination with free-field sound delivery in visual virtual reality, we tested participants in two auditory spatial tasks: front-back discrimination and 3D sound localization in front space. Front-back discrimination was easier for participants with symmetrical compared to asymmetrical hearing-loss, yet both groups reduced their front-back errors when head-movements were allowed. In 3D sound localization, free head-movements reduced errors in the horizontal dimension and in a composite measure that computed errors in 3D space. Errors in 3D space improved for participants with asymmetrical hearing-impairment when the head was free to move. These preliminary findings extend to aging adults with hearing-loss the literature on the advantage of head-movements on sound localization, and suggest that the disparity of auditory cues at the two ears can modulate this benefit. These results point to the possibility of taking advantage of self-regulation strategies and active behavior when promoting spatial hearing skills.

## Introduction

Age-related hearing loss (ARHL) is a major issue for individuals and the society. It develops gradually, often in a subtle fashion: at first it reduces the detection of high-pitched sounds and speech comprehension in noisy environments, then it progresses into a more generalized difficulty in understanding conversations (Davis, 2019). Although the impact of ARHL has been primarily investigated in relation to speech comprehension (Noble et al., 1995), it impacts auditory environment more broadly. In particular, it effects the ability to localize sounds in space, by changing binaural and monaural auditory cues available at each ear. Binaural cues (interaural level difference, ILD and interaural time difference, ITD) play a key role when determining the horizontal direction of sounds. In normal-hearing, sound localization exploits primarily low-frequency (< 1400 Hz) ITD cues, with a secondary role for high-frequency (>4000 Hz) ITD and ILD cues (see Macpherson and Middlebrooks, 2002). Monaural cues depend upon the direction of sounds with respect to the head and the external ear, and contribute primarily to front-back disambiguation, elevation estimation, and distance perception of sounds. They can be successfully extracted only from high-frequency sounds (>4000 Hz Middlebrooks, 2015). The high-frequencies loss of ARHL impacts high-frequency ITD and ILD cues, as well as monaural cues. Moreover, with declines in neural synchrony and reduced central inhibition related to advancing age, processing of auditory cues is hindered even more (Eddins et al., 2018). This results in worse front-back discrimination and impoverished localization of sounds on the vertical plane in elderly adults (Rakerd et al., 1998). Finally, in the case of asymmetric ARHL the imbalance between binaural cues could result in poor localization performances on the horizontal plane, considering the lack of high-frequency monaural cues that normally compensate for the inability of extracting binaural cues (Kumpik and King, 2019). In this context of reduced peripheral cues at the ears, how can aging adults improve their sound localization skills?

Head-movement during sound is a spontaneous and ubiquitous behavior that impacts on sound localization. Head-movements change the available auditory cues: rotations around the vertical axis modify ITD and ILD cues, whereas tilting the head impacts on monaural cues (Perrett and Noble, 1997a; Kato et al., 2003). Head-movements present several orientations of the ears to the sound and therefore provide richer and more dynamic auditory cues (Pollack and Rose, 1967). Although the role of head movements in spatial hearing has been advocated since the first half of the last century (Wallach, 1940), systematic investigations have only started in the last decades, also as a consequence of greater availability of motion-tracking technologies. Head-movements during sound improve sound localization in normal-hearing adults on both horizontal and vertical dimensions (Perrett and Noble, 1997a) and reduce front-back discrimination errors (Iwaya et al., 2003). In addition, head-movements improve sound localization in hearing-impaired adults (Brimijoin et al., 2012) and cochlear implant users (adults: Pastore et al., 2018; children: Coudert et al., 2022). If head-movements can improve sound localization in the context of ARHL remains, to the best of our knowledge, an open question.

This study aimed to examine if head-movements improve sound localization in aging adults with ARHL. Previous studies asked participants to perform stereotyped movements (e.g., keep their movements slow, continuous and in a ±30° range; Pastore et al., 2018) or forced passive head-movement through robotic control of the participant's head (Thurlow and Runge, 1967). Here, we opted for inviting participants to produce spontaneous head-movements while the sound was playing, without giving any specific instructions as to movement speed or extension (as in Coudert et al., 2022). To measure sound localization and head-movements, we exploited a visual virtual reality and motion tracking approach (Valzolgher et al., 2020a; Coudert et al., 2022), which allows extensive control over the audio-visual stimulation delivered to participants. We asked participants to localize sounds in a visual virtual reality scenario while recording their head-movements in real-time under two listening conditions: head-fixed and head free to move. Each participant performed two auditory spatial tasks: front-back discrimination and 3D sound localization in front space (participants responded using a hand-held tool and we measured their responses in azimuth, elevation and distance). We enrolled aged participants with different degrees of hearing-impairment, who were divided in two groups differing for hearing asymmetry: symmetrical and asymmetrical ARHL.

We expected spontaneous head-movements to facilitate sound localization in both tasks. Head-rotations around the vertical axis modify time of arrival and level of sounds at the two ears and therefore each degree of rotation is associated with different auditory binaural cues. Dynamical changes of binaural cues enable a more reliable selection between different possible sound sources that vary in 3D space (McAnally and Martin, 2014). Regarding front-back discrimination, rotations along the head vertical axis transform front-back confusion into left-right discrimination, increasing the possibility of using binaural cues. We expected a benefit in sound localization when head is free to move, particularly for asymmetrical ARHL participants who are more likely to experience auditory cues ambiguities during head-fixed listening.

## Materials and methods

### Participants

Sixteen participants (mean age 71, SD = 6.51, range = [61–82], 7 males) took part in the study. Sample size was driven by previous studies that investigated head-movements effects on sound localization in normal-hearing (Perrett and Noble, 1997b: *N* = 16) and hearing-impaired participants (Pastore et al., 2018: 7 listeners bilaterally implanted with cochlear implants, 5 of the patients with one implant turned off). Half of participants suffered symmetrical hearing-loss, with an average of 6.88 dB HL (SD = 3.23, range = [3–13]) difference in hearing threshold between the two ears. Hearing thresholds were 31.63 dB HL (SD = 8.94, range = [15–44]) in the worse ear, and 24.75 dB HL (SD = 9.85, range = [11–41]) in the best ear. The remaining half of participants suffered from asymmetrical hearing loss, with a difference in hearing threshold between ears of 39.38 dB HL (SD = 11.06, range = [25–60]). Hearing thresholds were 60.88 dB HL (SD = 7.94, range = [51–78]) in the worse ear, and 21.50 dB HL (SD = 7.31, range = [15–38]) in the best ear. All had normal or correct-to-normal vision. See Table 1 for further details.

**Table 1:** Personal and audiometric characteristics of participants.

**Participant**	**Group**	**Age**	**Sex**	**Worst ear**	**Threshold best ear**	**Threshold worst ear**	**Disparity between the two ears**
1	Asymmetric	68	M	Left	15 dB HL (normal)	63 dB HL (moderate severe)	48 dB HL
2	Asymmetric	69	M	Left	21 dB HL (slight)	58 dB HL (moderate)	37 dB HL
3	Asymmetric	77	M	Right	16 dB HL (slight)	58 dB HL (moderate severe)	42 dB HL
4	Asymmetric	71	F	Right	23 dB HL (slight)	51 dB HL (moderate)	28 dB HL
5	Asymmetric	81	F	Left	38 dB HL (mild)	63 dB HL (moderate severe)	25 dB HL
6	Asymmetric	62	M	Left	23 dB HL (slight)	60 dB HL (moderate severe)	37 dB HL
7	Asymmetric	63	M	Right	18 dB HL (slight)	78 dB HL (severe)	60 dB HL
8	Asymmetric	73	F	Left	18 dB HL (slight)	56 dB HL (moderate severe)	38 dB HL
9	Symmetric	65	F	Left	11 dB HL (normal)	15 dB HL (normal)^1^	4 dB HL
10	Symmetric	77	M	Left	29 dB HL (mild)	37 dB HL (mild)	8 dB HL
11	Symmetric	69	F	Left	22 dB HL (slight)	30 dB HL (mild)	8 dB HL
12	Symmetric	61	F	Right	18 dB HL (slight)	25 dB HL (slight)	7 dB HL
13	Symmetric	69	F	Left	16 dB HL (slight)	29 dB HL (mild)	13 dB HL
14	Symmetric	77	F	Left	33 dB HL (mild)	37 dB HL (mild)	4 dB HL
15	Symmetric	82	M	Left	41 dB HL (moderate)	44 dB HL (moderate)	3 dB HL
16	Symmetric	72	F	Right	28 dB HL (mild)	36 dB HL (mild)	8 dB HL

Personal and audiometric characteristics of participants. Classification criteria refer to Clark (1981). Uses and abuses of hearing loss classification. Asha, 23(7), 493–500. For each ear we tested frequencies at 125, 250, 500, 750, 1000, 1500, 2000, 3000, 4000, 6000, and 8000 Hz, through a pure tone audiometry.

Asymmetrical ARHL participants were recruited at the otolaryngology department of “S. Maria del Carmine” hospital in Rovereto (Italy), symmetrical ARHL participants were recruited through advertisement. All volunteers gave their informed consent before starting the experiment, which was approved by the Ethics Committee of the University of Trento (protocol number: 2019-037). The inclusion concerned only individuals without hearing aids, who did not use drugs, or reported a history of neurological or psychiatric problems. All participants completed the Montreal Cognitive Assessment test (MoCA, Italian version: Conti et al., 2015) to exclude possible cognitive decline and all obtained normal scores for their age.

### Stimuli

The auditory target was a white-noise (43–22000 Hz; sample rate: 44100 Hz), with an 80% amplitude-modulation at 2.5 Hz. We adopted this broadband stimulus to preserve processing of all frequencies available to each ear (Hofman et al., 1998; Savel et al., 2009; Gaveau et al., 2022; Valzolgher et al., 2022a). Moreover, we modulate noise's amplitude to facilitate ITD processing by reducing phase ambiguities (Macpherson and Middlebrooks, 2002). Sound was delivered at about 75 dB SPL, as measured from the participant's head using a decibel meter (TES1350A). Each auditory target lasted 5 seconds, to allow participants enough time to make spontaneous head-movements during the head-free condition. Auditory targets were delivered at pre-determined positions in each trial (see Procedure).

### Apparatus

The experiment was run using the HTC Vive system, a virtual reality and motion tracking device [see Valzolgher et al., 2020a]. This system comprised one Head-Mounted Display (HMD, resolution: 1080 × 1200 px, Field of View (FOV): 110°, Refresh rate: 90 Hz) for the presentation of visual stimuli; one hand-held tracker used by participants to collect pointing responses; one tracker placed above the speaker to monitor its position in real-time; one hand-held controller used by the experimenter to record the responses. Finally, two lighthouse base stations scanned the position of the HMD, the trackers and the controller in real-time. The HTC Vive system and the lighthouse base stations were controlled by a LDLC ZALMAN computer (OS: Windows 10 (10.0.0) 64bit; Graphic card: NVIDIA GeForce GTX 1060 6 GB; Processor: Intel Core i7-770 K, Quad-Core 4.2/4.5 GHz Turbo, Cache 8 Mo, TDP 95 W), using the Steam VR software and the development platform Unity.

All target sounds were delivered using a single real speaker (JBL GO Portable, 68.3 × 82.7 × 30.8 mm, Output Power 3.0 W, Frequency response 180–20 kHz, Signal-to-noise ratio >80 dB) at pre-determined positions within reaching space (see Procedure).

### Procedure

After a preliminary description of the experiment, participants sat on a rotating chair in the center of a room (3 × 4 × 5 m) and wore the HMD. They were immersed in a virtual empty room, with the exact same metrics as the real room in the laboratory. The rationale for showing a visual virtual environment was twofold: first, it allowed to eliminate all visual cues about the sound source position (i.e., the loudspeaker); second, it allowed to present participants with a visual environment that was devoid of furniture. A visible room is a more ecological compared to a fully dark environment during sound localization because it can provide visual references that guide acoustic space perception [Majdak et al., 2010; see also Valzolgher et al., 2020a]. In this virtual scenario, participants saw the tracker that they held in their hand, to allow more accurate pointing movements to the perceived sound source. Virtual reality has been used previously with elderly participants, with no harmful outcomes or stressful situations reported (Crespo et al., 2016). Likewise, no participant tested in the present work reported motion-sickness or discomfort.

Our experimental setup allowed to deliver sounds at pre-determined positions defined in head-centered coordinates at the beginning of each trial. Specifically, the system computed the pre-determined position in 3D space with respect to the center of the head and the interaural axis, and gave the experimenter visual cues (on a dedicated monitor) to guide the loudspeaker to the exact target position with a 5 cm tolerance (see Gaveau et al., 2022). The experimenter held the speaker in the target position for sound emission with her hand. Crucial to this procedure was the calibration of head position, which occurred each time the HMD was worn. This calibration was performed by marking with the experimenter's hand-held controller the position of the left and right ears of the participant.

After the participant familiarized with the virtual environment, two sound localization tasks were performed: front-back discrimination (see Front-back discrimination task) and 3D sound localization in front space (see 3D sound localization in front space task). Participants completed each task under two listening conditions: head-fixed or head free to move. During the head free condition, no suggestions were given regarding how to move the head and in this sense, movements represent a spontaneous strategy. The order of tasks and listening conditions were counterbalanced across participants. The entire procedure took 2 h on average, including preparation and pauses, with a VR immersion of 75 min.

Participants received instructions in the HMD to acquire a front facing posture at the beginning of each trial. Specifically, they saw in the HMD their head direction (indicated by a blue cross) and were instructed to align it with a white cross in the center of the virtual room. As soon as the two crosses were aligned, and the experimenter brought the loudspeaker to the pre-determined target position, the crosses disappeared and the sound was delivered. This approach allows participants to achieve a replicable sound target locations across trials, without using an external constraint (e.g., a chin-rest) which would have been incompatible with the free-head movement condition.

#### Front-back discrimination task

**Figure 1:**
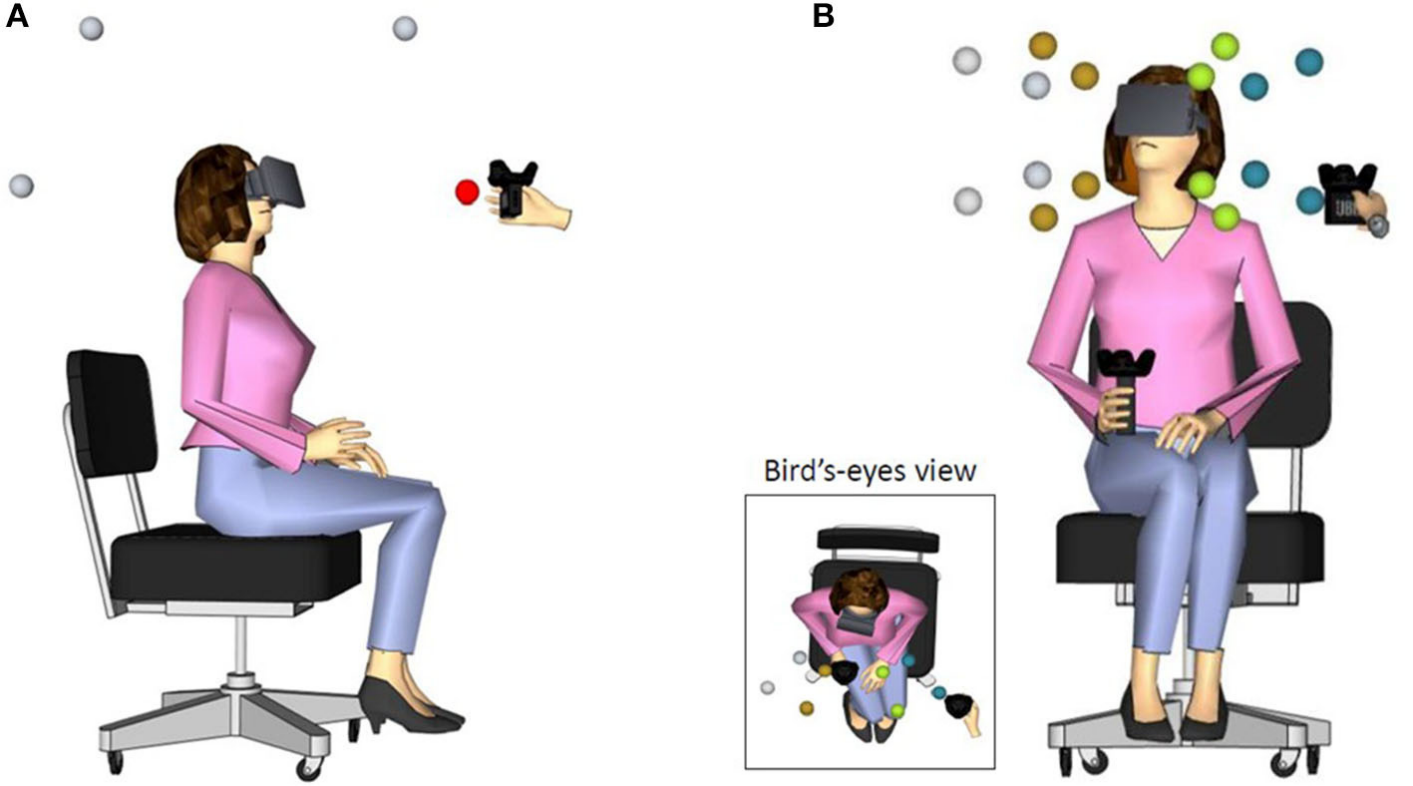
Schematic description of participant wearing the HMD in **(A)** the front-back discrimination task (lateral view) and **(B)** in the 3D sound localization in front space task (front and bird's-eye view). The spheres around the participant's head indicate the target positions. Different azimuth positions are marked by different colors only for illustrative purposes. The experimenter brought the tracked speaker (also shown in figure) at the pre-determined location identified in each trial.

In the front-back discrimination task, target positions were arranged along the participant's mid-sagittal plane, two at the front and two at the back (see Figure 1A). Specifically, the tracker connected to the loudspeaker was placed at 0° and 45° in front space, and at 135° and 180° in back space. All targets were delivered at about 50 centimeters from the center of the participant's head. Participants were instructed to listen to the sound and wait until its end before responding. They had to report verbally if the sound was emitted from front or back space. Responses were saved by the experimenter through the hand-held controller. No performance feedback was provided. Ten practice trials were included at the beginning of each block, to allow familiarization with the procedure. A total of 32 trials (8 repetitions for each of the 4 positions) were presented in randomized order within each listening condition.

#### 3D sound localization in front space task

In the 3D sound localization task, target positions were in front space, at four azimuth (± 40° and ± 20° with respect to the midsagittal plane), two elevation (– 25° and 15° with respect to the plane passing through the ears) and two distances (35 or 55 centimeters from the center of the participant's head; see Figure 1B). Participants listened to the sound and wait until its end before responding. During sound emission, they kept their right hand holding the tracker stationary at the chest. When the sound ended, they were instructed to move the controller to the perceived position of the sound and validate their response by pressing a button on the tracker. Then, the experimenter triggered the beginning of the following trial. No performance feedback was provided [for a similar procedure see also Valzolgher et al., 2020a]. Ten practice trials were included at the beginning of each block, to allow familiarization with the procedure. A total of 48 trials (4 repetitions for each of the 16 positions) were presented in randomized order within each listening condition.

### Analysis

All data were analyzed using Linear Mixed Effects (LME) or Generalized Linear Mixed Effects (GLME) models in R studio with the packages lme4 (Bates et al., 2014), car (Fox and Weisberg, 2020), and lmerTest (Kuznetsova et al., 2017). When appropriate, we corrected the skewness of distributions by log-transforming the variables. The raw data can be retrieved from osf.io/57chk. Details of kinematic analyses could be found in Supplementary Results.

To analyze the performance, we measured error rates for front-back discrimination task and average 3D errors for sound localization in front space. 3D errors represent the distance in centimeters between perceived positions of sources and the actual speaker's location. We then analyzed the average error on azimuth (in degrees), elevation (in degrees), and distance (in centimeters).

## Results

As instructed, participants refrained from moving the head in the head-fixed condition and made spontaneous head-movements in the head-free condition. Occasional trials with head-movements in the head-fixed condition were removed from the analyses (0.29% of trials in the front-back discrimination task; 1.37% of trials in the 3D sound localization task). In the front-back discrimination task, during the head-free condition, participants made 4.0 (SD = 1.9) spontaneous head-movements, with a horizontal head-rotation extent of 46.8° and a vertical head-rotation extent of 18.6°. Instead, in the 3D localization task, they made 3.3 (SD = 1.5) spontaneous head-movements, with a horizontal head-rotation extent of 37.8° and a vertical head-rotation extent of 24.2°.

**Figure 2:**
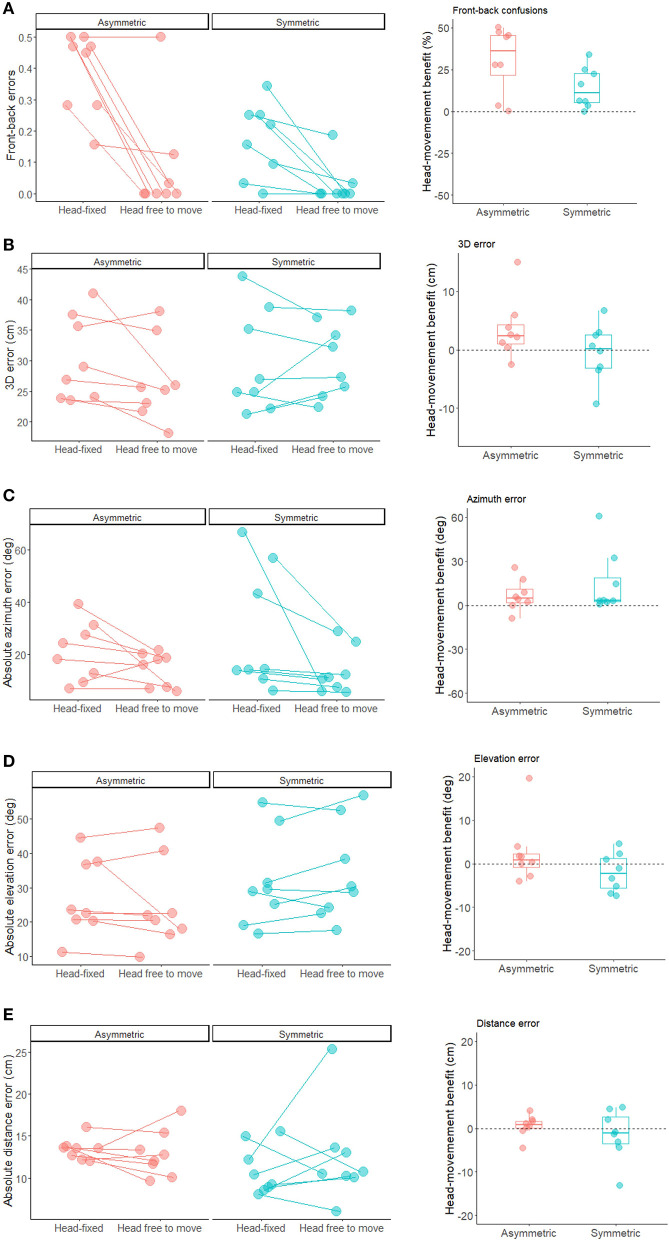
Individual performance (left plots) and head-movement benefit (right plots) for each participant in the front-back task **(A)** as well as the 3D sound localization task **(B)** 3D error; **(C)** azimuth error; **(D)** elevation error; **(E)** distance error. The head-movement benefit was calculated as the difference between errors in the head-free vs. head-fixed listening condition. Positive values indicate better performance when the head was free to move.

To study the effect of listening condition on front-back discrimination performance, we entered the binomial responses of each participant in a GLME model (family = binomial), using listening condition and group as categorical fixed effects and the participants' intercept as a random effect. Percent errors in front-back discrimination were smaller for symmetrical (9.7% ± 10%) compared to asymmetrical hearing-loss participants (23.5% ± 11.8%; main effect of group: X^2^(1) = 9.11, *p* = 0.003). Importantly, spontaneous head-movements reduced percent errors (5.5% ± 13.0%) compared to the head-fixed condition (27.8% ± 16.6%) for both groups (main effect of listening condition: X^2^(1) = 58.98, *p* < 0.001; see Figure 2A).

To study the 3D sound localization in front plane task, we computed the distance in centimeters between the 3D position of the sound source indicated in each trial and the actual 3D location of the speaker (i.e., 3D error). Trials in which participants moved the controller held in their hand during sound emission were rejected (2.0%). Additionally, 12.4% deviant data-points were excluded from the analyses following quantile-to-quantile plot inspection. We entered the 3D error in LME model, using listening condition and group as categorical fixed effects and the participants' intercept as a random effect. Spontaneous head-movements reduced the 3D error for participants with asymmetrical hearing-loss, whereas this benefit was not evident in participants with symmetrical hearing-loss resulting in a significant two-way interaction (X^2^(1) = 16.32, *p* < 0.001; see Figure 2B and Table 2). The main effect of listening condition also reached significance (X^2^(1) = 20.63, *p* < 0.001), but subsidiary to the higher order interaction.

**Table 2:** Effects of spontaneous head-movements on symmetrical and asymmetrical ARHL participants.

	**Symmetrical**		**Asymmetrical**	
	**Head free to move**	**Head static**	**Head free to move**	**Head static**
**3D sound localization in front space**				
Azimuth	13.38 (± 8.79)	28.24 (± 23.70)	14.39 (± 6.60)	21.19 (± 11.35)
Elevation	33.85 (± 14.16)	31.81 (± 13.51)	24.65 (± 12.72)	27.12 (± 11.13)
Distance	12.43 (± 6.67)	10.94 (± 2.94)	12.85 (± 2.78)	13.41 (± 1.25)
3D error	30.15 (± 6.91)	29.73 (± 8.37)	26.58 (± 6.68)	30.15 (± 6.91)
**Front-back discrimination**				
Error rate	0.03 (± 0.07)	0.17 (± 0.12)	0.08 (± 0.17)	0.39 (± 0.13)

Effects of spontaneous head-movements on symmetrical and asymmetrical ARHL participants. Means with standard deviation in parenthesis. Note that azimuth and elevation errors are expressed in degrees, distance and 3D error in cm.

We also examined the effect of listening posture on absolute localization errors in each dimension separately (i.e., azimuth, elevation and distance; see Table 2), using a LME models similar to the one adopted for the 3D error. Quantile-to-quantile plot inspection led to exclusion of 7.9% for azimuth, 6.4% for elevation, and 6.1% for distance. For azimuth (see Figure 2C), we found a main effect of listening condition (X^2^(1) = 32.92, *p* < 0.001), caused by smaller absolute errors in the head-free (13.8°± 7.9°) compared to the head-fixed condition (25.6° ± 21.2°). For elevation, we also found a main effect of listening condition (X^2^(1) = 5.32, *p* < 0.02) and a two-way interaction (X^2^(1) = 7.16, *p* = 0.007), caused by larger benefits of head-movements for participants with asymmetrical than symmetrical hearing-loss (Figure 2D). Finally, for distance, no main effect or interaction emerged (all *p*-values > 0.21; Figure 2E).

While the main purpose of our experiment was to examine effects of head-movements on sound localization performance, in Supplementary Results we also report our analyses on head-movements during the head free to move condition.

## Discussion

In the present study, we examined if spontaneous head-movements can improve sound localization in aging adults with symmetrical or asymmetrical ARHL. We examined the ability to discriminate between sounds presented from front and back space and the ability to localize 3D sounds in front space under two different listening conditions: head-fixed and head free to move during sound emission.

Our findings show that spontaneous head-movements during sound presentation reduce front-back error and facilitate 3D sound localization in front space. The latter effect was more consistent for participants with asymmetrical hearing-loss. This result is coherent with previous studies that investigated the benefits of head-movements on front-back discrimination in young adults with normal-hearing (Perrett and Noble, 1997a,b; Iwaya et al., 2003) or with hearing-impairment (Mueller et al., 2014; Brimijoin and Akeroyd, 2016). Similarly, it corroborates the benefit of head-movements for sound localization in front space reported for young adults with normal-hearing (Brimijoin et al., 2013; Morikawa and Hirahara, 2013) or hearing-impairment (Coudert et al., 2022).

Our study extends to aging adults with ARHL the literature on the advantage of head-movements on sound localization. Although it has been documented that sound localization abilities decrease with advancing age (Dobreva and O'Neill, 2011), previous studies have mostly adopted a static-head approach when examining aging participants. To the best of our knowledge, the only exception to this wide-spread approach is represented by a study by Otte et al. (2013), in which they registered comparable localization performance in the horizontal dimension in young and older adults. Participants were exposed to an open-loop head-movement localization paradigm with sound sources varying horizontally and on the vertical plane. Target sounds were set to last 150 ms, precisely to ensure that the head-saccades toward the sound “always started after stimulus offset, which denied listeners potential acoustic feedback during their response” (Otte et al., 2013; p. 264). Yet, participants were free to move their head during the task and encoded sound position within a reference-frame that served head-movement. Although Otte et collaborators allowed head-movement in aging adults, the stimuli were too short to allow active listening experience (i.e., moving the head during the sound emission). Furthermore, the authors did not compare older adults' performance during head-fixed vs. free to move condition.

In the present study, we compared directly the two listening conditions. We did not manipulate directly the possibility of exploiting binaural and monaural cues by altering sounds frequency. However, during the active listening condition, participants were free to explore the acoustic space as they wanted, for a relatively long period of time (5 s). In the following paragraph we discuss the possible reasons subtending benefit of spontaneous head movement while listening.

The first set of explanation is related to the more peripheral consequences of moving the head. Wallach was the first to suggest that head rotations along the vertical axis can reduce the “cone of confusion” by 50% during front-back discrimination of sounds (Wallach, 1940). Wightman and Kistler (1999) proved that this benefit can be experienced even when sound sources change position while listeners maintain a static head-posture. This suggests that head-movement benefits may partly reflect the richer auditory cues available to the ears as the peripheral input becomes dynamic (see also Thurlow and Runge, 1967; Perrett and Noble, 1997a,b; Kato et al., 2003; McAnally and Martin, 2014). In this perspective, ARHL participants tested in this study struggled to discriminate front-back in head-fixed listening condition, likely as a consequence of their impoverished monaural spectral cues. Participants could have benefited from head-movements because turning the head changed front-back discrimination from a purely monaural task, to a task that could be solved exploiting binaural auditory cues. Another possibility is that moving the head introduced greater dynamicity in the monaural cues available at the ears. In support of this second, additional, interpretation we observed that asymmetrical ARHL participants improved in the 3D sound localization in front space task specifically in the vertical dimension. In other words, it appears that when localizing sounds head-movements allowed them to better exploit the monaural auditory cues needed for discriminating sound position in elevation.

In addition to these explanations based on changes occurring at the peripheral level, it is important to consider that head-movements are a paradigmatic example of active listening and can also reflect self-regulating strategies. In this respect, the interpretation of any head-movement related benefit becomes more cognitive, i.e., related to predictive behaviors that participants put in place when aiming to solve perceptual uncertainties. For asymmetrical ARHL participants, turning the head may have been an intentional strategy to exploit the head-shadow effect, maximizing sound intensity at the best ear (see also Valzolgher et al., 2020b, 2022b).

In conclusion, our findings extend to ARHL the literature on the advantage of head-movements on sound localization, and provide initial evidence that this benefit may be influenced by the disparity of auditory cues at the two ears. These results could be exploited when planning specific interventions in different hearing-impaired populations, and particularly point to the possibility of taking advantage of both acoustic benefit of head-movements and active behavioral strategies when promoting spatial hearing skills. Moreover, the present study underlines the importance of promoting more ecological scenarios, in order to consider active listening conditions during the evaluation of auditory abilities.

Given the limited number of participants enrolled in this research our findings provide only preliminary evidence. Future studies should examine the effect of active listening across different severity of hearing loss, in large scale studies. They may also compare participants with similar hearing-impairment but different age (young vs. aged) to examine the possible contributions of aging on the observed effects. Furthermore, it would be important to test the effect of moving the head as a function of sound features, and particularly examine these effects with more ecological sounds (e.g., speech).

Finally, although in the present study head-movements were implemented spontaneously, active head-orienting to sounds could be trained. Studies in this direction have already been conducted in normal hearing young adults with one ear plugged, to simulate a unilateral hearing loss condition (i.e., Valzolgher et al., 2020b, 2022b) and in bilateral cochlear implant users (Valzolgher et al., 2022a). A relevant future direction for research would be to test training paradigms to promote effective behavioral strategies during sound localization even in ARHL.

## Data availability statement

The datasets presented in this study can be found in online repositories. The names of the repository/repositories and accession number(s) can be found below: osf.io/57chk.

## Ethics statement

The studies involving human participants were reviewed and approved by Research Ethics Committee, University of Trento (2019–037). The patients/participants provided their written informed consent to participate in this study. Written informed consent was obtained from the individual(s) for the publication of any potentially identifiable images or data included in this article.

## Author contributions

EGe, DS, GF, and FP contributed to conception and design of the study. EGe, GV, and CV organized the database. EGe, FP, and CV performed the statistical analysis. EGe and CV wrote the first draft of the manuscript. EGe, EGi, AF, FP, and CV wrote sections of the manuscript. All authors contributed to the article and approved the submitted version.

## Funding

The author(s) disclosed receipt of the following financial support for the research, authorship, and/or publication of this article: FP was supported by a grant of the Agence Nationale de la Recherche (ANR-16-CE17-0016, VIRTUALHEARING3D, France), by a prize of the Foundation Medisite (France), by the Neurodis Foundation (France), and by a grant from the Italian Ministry for Research and University (MUR, PRIN 20177894ZH). CV was supported by a grant of the Università Italo-Francese/Université Franco-Italienne, the Zegna Founder's Scholarship and Associazione Amici di Claudio Demattè. EGe, FP, and CV were supported by a grant of the Velux Stiftung (n. 1439). FP, CV, and AF were supported by a grant of the Agence Nationale de la Recherche (ANR-16-CE17-0016, VIRTUAL- HEARING3D, France) and by a prize of the Fondation Medisite (France). The study was supported by the IHU CeSaMe ANR-10-IBHU-0003 and it was performed within the framework of the LABEX CORTEX (ANR-11- LABX-0042) of Université de Lyon.

## Conflict of interest

The authors declare that the research was conducted in the absence of any commercial or financial relationships that could be construed as a potential conflict of interest.

## Publisher's note

All claims expressed in this article are solely those of the authors and do not necessarily represent those of their affiliated organizations, or those of the publisher, the editors and the reviewers. Any product that may be evaluated in this article, or claim that may be made by its manufacturer, is not guaranteed or endorsed by the publisher.
