# Supplementary material for: Spontaneous head-movements improve sound localization in aging adults with hearing loss
Source: Front Hum Neurosci. 2022 Oct 13;16:1026056. doi: 10.3389/fnhum.2022.1026056 (PMC9609159; doi:10.3389/fnhum.2022.1026056)
Supplement: Supplementary file 1 [file Data_Sheet_1.docx]

Supplementary Results

The main purpose of our experiment was to examine effects of head-movements on sound localization performance. However, here we also present and discuss head behavior during the front-back discrimination task and the 3D sound localization task. All analyses below include only the listening condition in which the head was free to move. Recall that all head-movements were spontaneous.

Kinematic analyses

To examine head behavior, we focused on the number of movements as well as the extent of head-rotations around the vertical and horizontal axis within each trial. Measures of head-kinematic were obtained using a custom-made software for motion tracking analysis developed at the IMPACT team of the Centre de Recherche en Neuroscience de Lyon (CRNL, Lyon, France), running on MATLAB R2019b (see Gaveau et al., 2022). We calculated tangential velocity on x, y, and z axis (in degrees of rotation) using a two-point central difference derivate algorithm (Bahill & McDonald, 1983) with five points for the half-window. The onset and the end of head- and hand-movements were computed automatically using a velocity-based threshold (10°/s) and were checked manually by visualizing the spatial rotation changes of head and hand and their speed. We were able to establish the spatial-temporal profile of head and hand behaviors and extract relevant parameters for subsequent analyses.

Front-back discrimination task

To study head-related behavior during the front-back discrimination task, we considered number of head-movements, extent of head-rotations around the vertical axis (left-right rotations) and extent of head-rotations around the horizontal axis (up-down rotations).

We entered number of head-movement in a GLMER (family = Poisson) with target position (front, back) and group (symmetrical, asymmetrical ARHL participants) as fixed effects and the participants’ intercept as a random effect. We did not observe any effect of sound source position, group or interaction (all *ps* > 0.14).

A similar analysis was conducted on extent of head-rotation around vertical axis using a LMER model. We observed head-rotations of greater extent when the sound was emitted from the back (main effect of target position: $X^{2}\left( 1 \right)=7.23, p=0.007)$. The main effect was further qualified by the two-way interaction between target position and group ($X^{2}\left( 1 \right)=23.45, p<0.001)$, that showed greater extent of head-rotation for back sounds in the symmetrical ARHL group (see Figure S1). A similar analysis on extent of head-rotation around the horizontal axis did not reveal any effect of target position, group or interaction (all *ps* > 0.20).

These results suggest a possible effect of listening experience onto the implementation of head-movement strategies, as we registered different extent of head-rotation around vertical axis (Y axis) in the two groups, as a function of sound source locations. Differences in head-movements behaviors might explain differences in performance for the front-back discrimination task we have observed during the head-free condition, even if performance of both groups are near ceiling during this condition (see Figure 2 and main text). When considering head-movement data and performance errors together, it seems that although both groups significantly improved when they could move their heads, the subtending head-extent exploration differed for the target emitted from the back. However, these results should be considered preliminary as further studies are needed to deeply investigate the implementation of such behaviors based in aged participants with asymmetric hearing deficits.


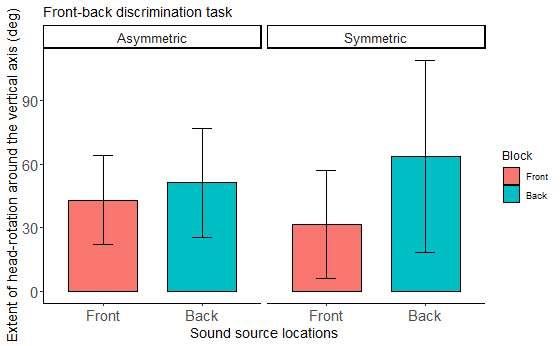


**Figure S1.** Extent of head-rotation around the vertical axis in the front-back discrimination task, as a function of group and sound source position.

**3D sound localization in front space**

To study head-related behavior during the 3D sound localization task, we entered number of head-movement in a GLMER (family = Poisson) with target position in azimuth (-40, -20, 20, 40) and group (symmetrical, asymmetrical ARHL participants) as fixed effects and the participants’ intercept as a random effect. We recorded sound locations in azimuth for all asymmetrical ARHL participants so that target positions in the space ipsilateral to the ear with the higher hearing threshold were coded as left targets. No main effect emerged (all *ps* > 0.40), but group by target position interaction was significant ($X^{2}\left( 3 \right)=8.61, p=0.03)$. While for asymmetrical ARHL participants number of movements did not vary as a function of target, for symmetrical ARHL we observed that number of movements peaked when the target was at -20° (with a significant difference with respect to 40° only). This result was not expected and will not be discussed further (Figure S2A).

A similar analysis was conducted on the extent of head-rotations around the vertical axis. Again, no main effect emerged (all *ps* > 0.32), but the group by target position interaction reached significance ($X^{2}\left( 3 \right)=18.31, p<0.001$). For asymmetrical ARHL participants extent of head-rotations did not vary as a function of target position, whereas for symmetrical ARHL extent of head-rotations was higher for peripheral as compared to central sounds (see Figure S2B). Furthermore, a similar analysis on head-rotation bias around the vertical axis, that is the sum of values of the rightward and leftward head-rotation extremity (Valzolgher et al., 2022), was performed. This analysis revealed a main effect of target position ($X^{2}\left( 3 \right)=163.33, p<0.001$), and the two-way interaction between group and target position ($X^{2}\left( 3 \right)=14.17, p=0.003$). For symmetrical ARHL participants head-rotation bias was different for each target position, for asymmetrical ARHL the bias did not differ between -40° and -20° and between 20° and 40°. Furthermore, we also noticed that bias measured when sound was emitted by target position 40° was higher for symmetrical as compared to asymmetrical ARHL participants, as visible in Figure S2C.

These results highlight that the comparable performance in azimuth (as we did not find an effect of group on absolute errors in azimuth plane for the head free to move listening condition) is although related to different head-movement behaviors of the two-groups. In particular, symmetrical ARHL participants increased the exploration of peripheral target positions and orient their head in the hemisphere where the sound source location was positioned (as indicated by the bias in head extensions). On the contrary, participants with asymmetrical ARHL did not increment the extent of their rotation based on target positions in space. Interestingly, head-rotation bias suggested that asymmetrical ARHL participants turned their head toward the side of the best ear (right) when the target was emitted from this side with a lower extent as compared to symmetrical ARHL. Similarly, we observed (only numerically) that they turn their head toward worse ear (left) with a greater extent when sound come from this side as compared to symmetrical ARHL. Furthermore, as visible in Figure S2 (C), in the asymmetrical ARHL group the rotation bias toward the worst ear (left) for -40° and -20° target positions has greater extent in absolute values as compared to the bias toward the best ear (right) for 20° and 40° sound source positions. Symmetrical ARHL participants show a bias symmetrically distributed between the two sides of space, whereas the asymmetrical ARHL group seems to have a more prominent bias toward the best ear (right). These data might reflect head-movement strategies for approaching the better ear to sound sources, but further studies are required to investigate this hypothesis further.


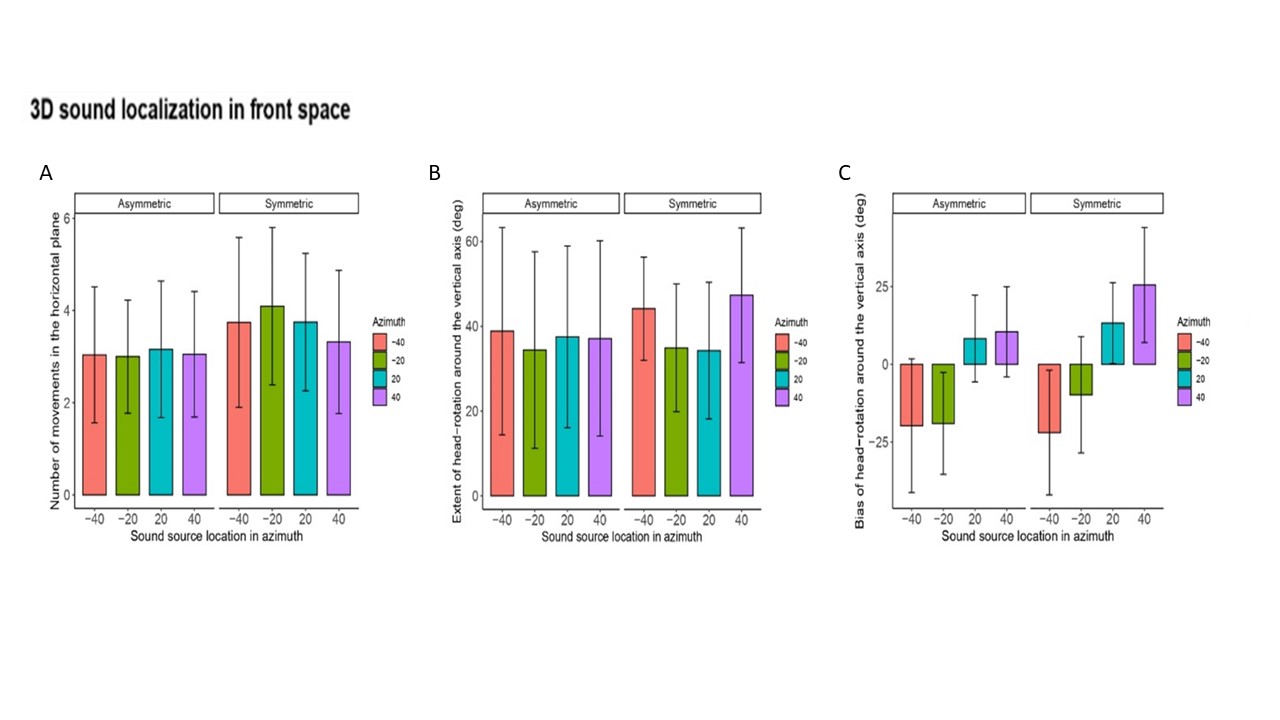


**Figure S2.** Number of head-movements in the horizontal plane depending on target positions in azimuth (A); extent of head-rotation around the vertical axis depending on target positions in azimuth; (B); bias of head-rotations around the vertical axis depending on target positions in azimuth (C).

**References**

Bahill, A. T., & McDonald, J. D. (1983). Smooth pursuit eye movements in response to predictable target motions. Vision research, 23(12), 1573-1583.

Gaveau, V., Coudert, A., Salemme, R., Koun, E., Desoche, C., Truy, E., Farnè, A. & Pavani, F. (2022). Benefits of active listening during 3D sound localization. Experimental Brain Research, 1-17.

Valzolgher, C., Todeschini, M., Verdelet, G., Gatel, J., Salemme, R., Gaveau, V., Gatel, J., Salemme, R., Gaveau, V., Truy, E., Farnè, A. & Pavani, F. (2022). Adapting to altered auditory cues: generalization from manual reaching to head pointing. PloS one, 17(4), e0263509.
